# Polymorphism in glassy silicon: Inherited from liquid-liquid phase transition in supercooled liquid

**DOI:** 10.1038/srep08590

**Published:** 2015-02-26

**Authors:** Shiliang Zhang, Li-Min Wang, Xinyu Zhang, Li Qi, Suhong Zhang, Mingzhen Ma, Riping Liu

**Affiliations:** 1 State Key Laboratory of Metastable Materials Science and Technology, Yanshan University, Qinhuangdao 066004, China; 2 School of Science, Yanshan University, Qinhuangdao 066004, China

## Abstract

Combining molecular dynamics (MD) simulation and Voronoi polyhedral analyses, we discussed the microstructure evolution in liquid and glassy silicon during cooling by focusing on the fraction of various clusters. Liquid-liquid phase transition (LLPT) is detected in supercooled liquid silicon However, freezing the high-density liquid (HDL) to the glassy state is not achieved as the quenching rate goes up to 10^14^ K/s. The polyamorphism in glassy silicon is found to be mainly associated with low-density liquid (LDL).

The liquid-liquid phase transition (LLPT) is a first-order transition between two liquids with distinct densities, a high-density liquid (HDL) and a low-density liquid (LDL)^1^,^2^. The unusual behaviors have been reported in some substances suh as supercooled water^3^,^4^,^5^,^6^, liquid carbon^7^,^8^, liquid phosphorus^9^, liquid nitrogen^10^ and supercooled silicon^11^,^12^,^13^. Silicon was predicted to have a phase transition below the freezing point by Aptekar^14^ in 1979. Sastry and Angell presented thermodynamic evidence of LLPT via molecular dynamics simulation in 2003^11^. Using X-ray diffraction experiments and *ab-initio* molecular dynamics, Jakse *et al*^15^ found that the coordination number reduces on supercooling, strongly implying the occurance of the LLPT in the supercooled silicon. Recently, a number of studies have been explored for the LLPT in supercooled silicon focusing on the electronic density of states^16^, dynamics and structural evolution^17^,^18^, valence electrons evolution^19^, perturbations^20^, negative pressure^21^. The main features involved in the LLPT of supercooled silicon are (i) the reduction of average coordination number from high coordination (above 5^11^,^21^) to low coordination (about 4^11^,^13^,^21^); (ii) the decrease in number density from 0.053 to 0.050 atoms/Å^3^ 13; (iii) the decrease in diffusivity by roughly two orders of magnitude^11^,^13^; (iv) the transition from metal-like to semiconductor-like behaivors^16^,^22^. However the relationship between the microstructure of silicon melts and the LLPT remains to be clarified.

Glasses are generally regarded as “frozen” liquids, and a glass could retain the configurational features of the liquid during quenching with certain short-range order (SRO). The medium-range order (MRO) is also argue in glasses with a correlation length extending the first peak in pair coorelation functions (PCF) to a distance up to 1 ~ 2 nm, making the glass different from the liquid.

Since glasses are in non-equilibrium states, and different configurations could be achieved during structural relaxation^23^,^24^,^25^. In recent years, a discontinous structural (or volumetric and enthalpic) change has been observed in some glasses, directly leading to the identification of polyamorphism. Polyamorphism in glassy silicon has been argued in some models^26^. Common to the models is the two distinct states of a metallic and a semiconducting glasses corresponding to low-density amorphous (LDA) and high-density amorphous (HDA) silicon^27^. Recently, Giovambattista *et al* reported^28^ that LLPT is necessary for the phase transition between the LDA and HDA in water, suggesting a possible link between the glass polyamorphism transition (GPT) and LLPT. In this work, we report the studies of the LLPT and the glass polyamorphism in supercooled silicon via molecular dynamics (MD) simulation.

## Results

### The evidence of LLPT

**Figure 1:**
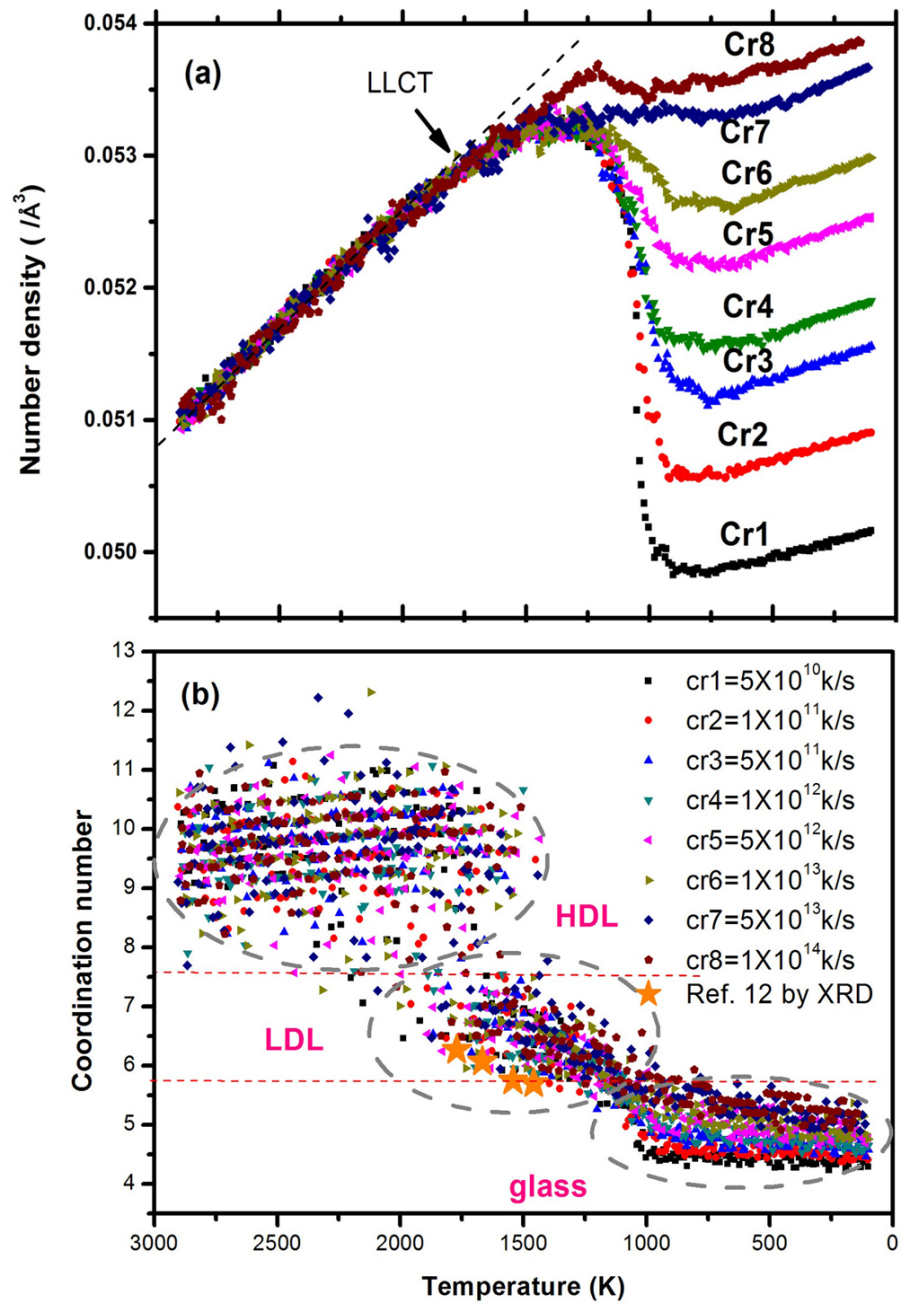
Dependence of the number density and first neighbor coordination on cooling rate in silicon. In (a), the dramatic shift in density begins between 1800 K and 1500 K, showing the liquid-liquid phase transition (LLPT) in the supercooled silicon. In (b), three regions is well defined from the coordination number, showing high density liquid (HDL), low density liquid (LDL) and glass. The orange star is the results from X-ray diffraction experiments reported in Ref. 12.

Figure 1(a) shows the temperature dependence of number density. One can see that the slope of density begins to shift between 1800 K and 1500 K, indicating the density change in the supercooled silicon during the cooling process. Figure 1(b) shows the coordination number distribution in the temperature range. It is seen that above ~1550 K, the coordination number is mainly in the range of 7.5 ~ 11.5 with a fluctuation of about 3, suggesting that particle fluctuation was considerable in the high temperature liquid. Below the melting point 1830 K, the fluctuation of the coordination number decreases from 3 to 2 from about 1550 to 980 K and the coordination number changes from 7.5 to 5.5. The marked drop of the coordination number suggests the changes in the liquid structure, implying the possible occurance of LLPT in supercooled silicon. Below 980 K, an abrupt change can be seen from Figure 1(a) and (b). The coordination number is around 5.5–4.5, and the transition from liquid to solid is complete. The three regions observed here by the coordination number are similar to the results reported in other groups^11^,^13^,^16^,^21^, and are consistent with the the X-ray diffraction experiments^12^.

**Figure 2:**
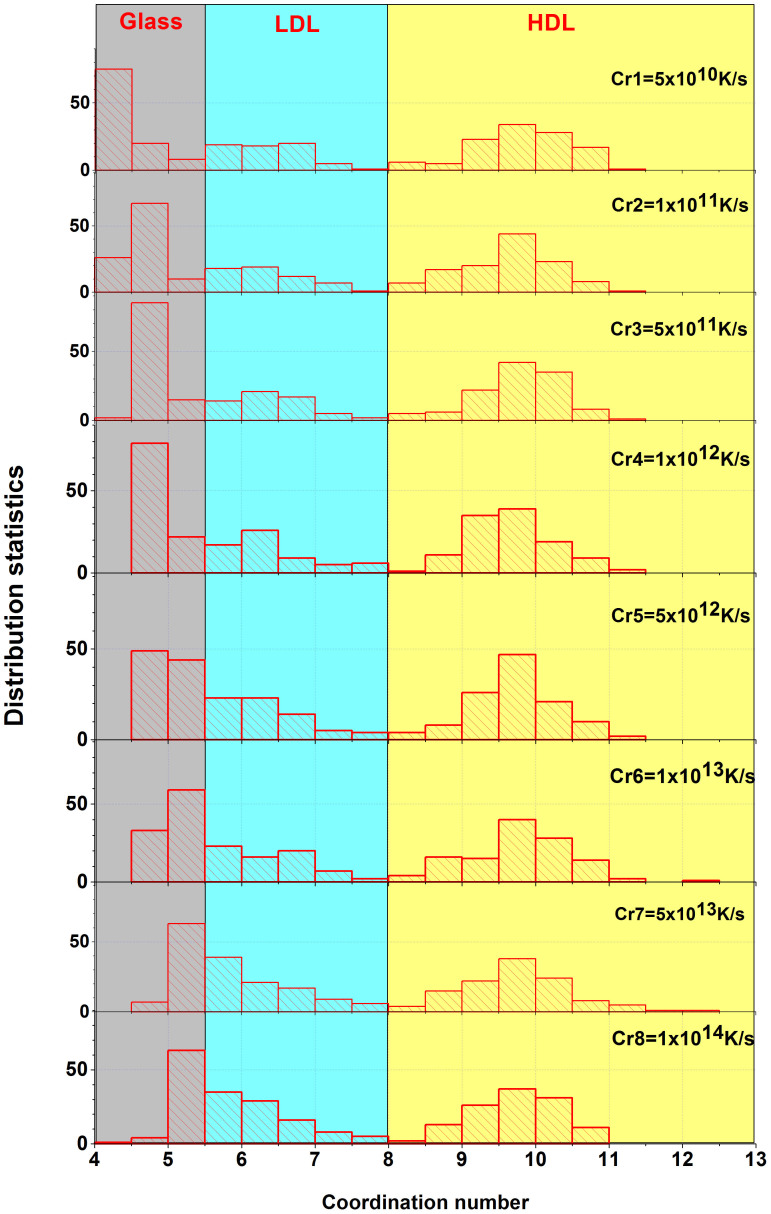
Dependence between the coordination number distribution and cooling rate. The gray area (coordination number varying from 4 to 5.5), light cyan area (coordination number from 5.5 to 8) and light yellow area (coordination number from 8 to 13) define the structure of the glass, LDL and HDL. The distributions in HDL are relatively stable, while the distributions in LDL and glass vary.

Figure 2 shows the relationship between the coordination number distribution and cooling rate. In the relative high coordination number region where the HDL structure has a coordination number of 9.5–10. The distributions are similar to Gaussian function and does not show a strong dependence on cooling rate. In the middle coordination number region, the distributions differ much with different cooling rate. For the high cooling rate, the coordination number 5.5–6 became dominant. In the low coordination number region, the coordination number changes from 4–4.5 to 5–5.5. It appears that the difference of the coordination numbers depends on cooling rate.

**Figure 3:**
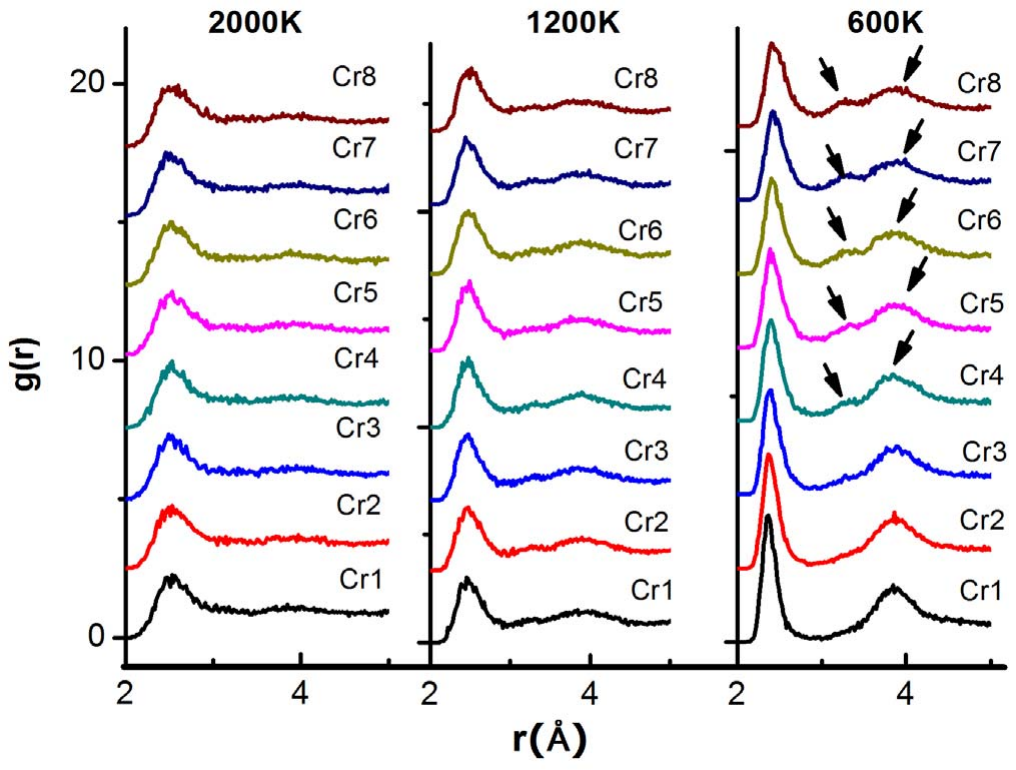
Pair correlation functions of the silicon structures at three temperatures, representing high density liquid, low density liquid and glass. The arrows of the 600 K curve indicate the split of the second peaks.

Base on the results in Figure 2, three typical liquid at 2000 K, 1200 K and 600 K, are selected for the structural analyses to represent the liquid, supercooled liquid and glassy silicon. Figure 3 shows the raw structure information of the three structures with different cooling rates. The comparison of the 2000 K and 1200 K liquids tells the resolved change in the profiles of the first peaks. The peaks become sharp significantly during cooling with the decrease in width and the increse in height. It indicates the basic difference in the SRO between the 2000 K and 1200 K liquids.

**Figure 4:**
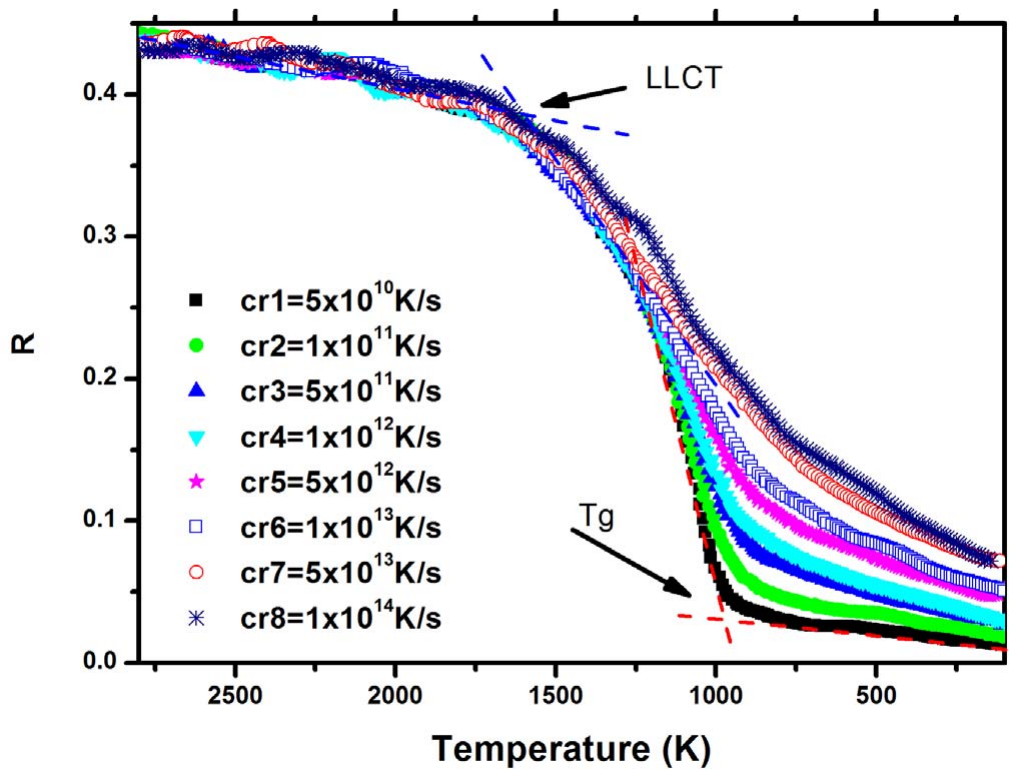
Temperature of Abraham ratio (R = g_min_/g_max_) for various cooling rates. The inflexions define two types of phase transitions.

Abraham parameer, R = g_min_/g_max_,^29^, is usually used to describe the change of SRO, especially to identify the glass transition during cooling. Figure 4 showed the dependence of the Abraham ratio R on temperature and cooling rate. Two inflexions can be seen during the cooling process. Near 950 K, the glass transition temperature *T_g_* is easily identified, and agrees well with the other simulation and experimental results^27^. At about 1550 K, the inflexion suggests a SRO transition in supercooled liquid silicon. Based on the results of the coordination number, density and potential energy showed above, the transition temperature could correspond to liquid-liquid phase transition critical temperature (LLCT). This value is larger than Sastry's classic molecular dynamics simulation result of 1060 K^11^, also larger than Ganesh's first principles molecular dynamics result of 1232 K^16^, and somehow close to the result of 1600 K (*P* = −0.5 GPa) via viscosities calculation method predicted by Deb *et al*^30^. The difference might partly come from the impact of the cooling rate.

### Structure transitions in LLPT

**Figure 5:**
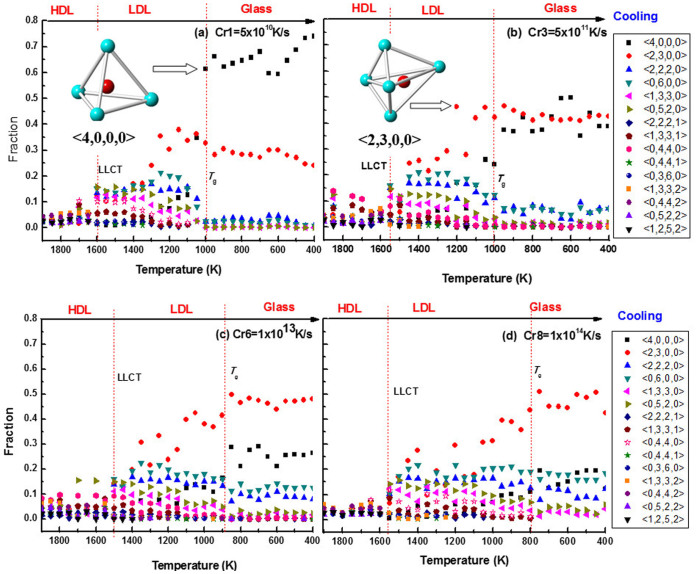
Population of Voronoi clusters in supercooled silicon quenched from four cooling rates. The dominant clusters of <4, 0, 0, 0> and <2, 3, 0, 0> is shown in the insets of (a) and (b).

**Figure 6:**
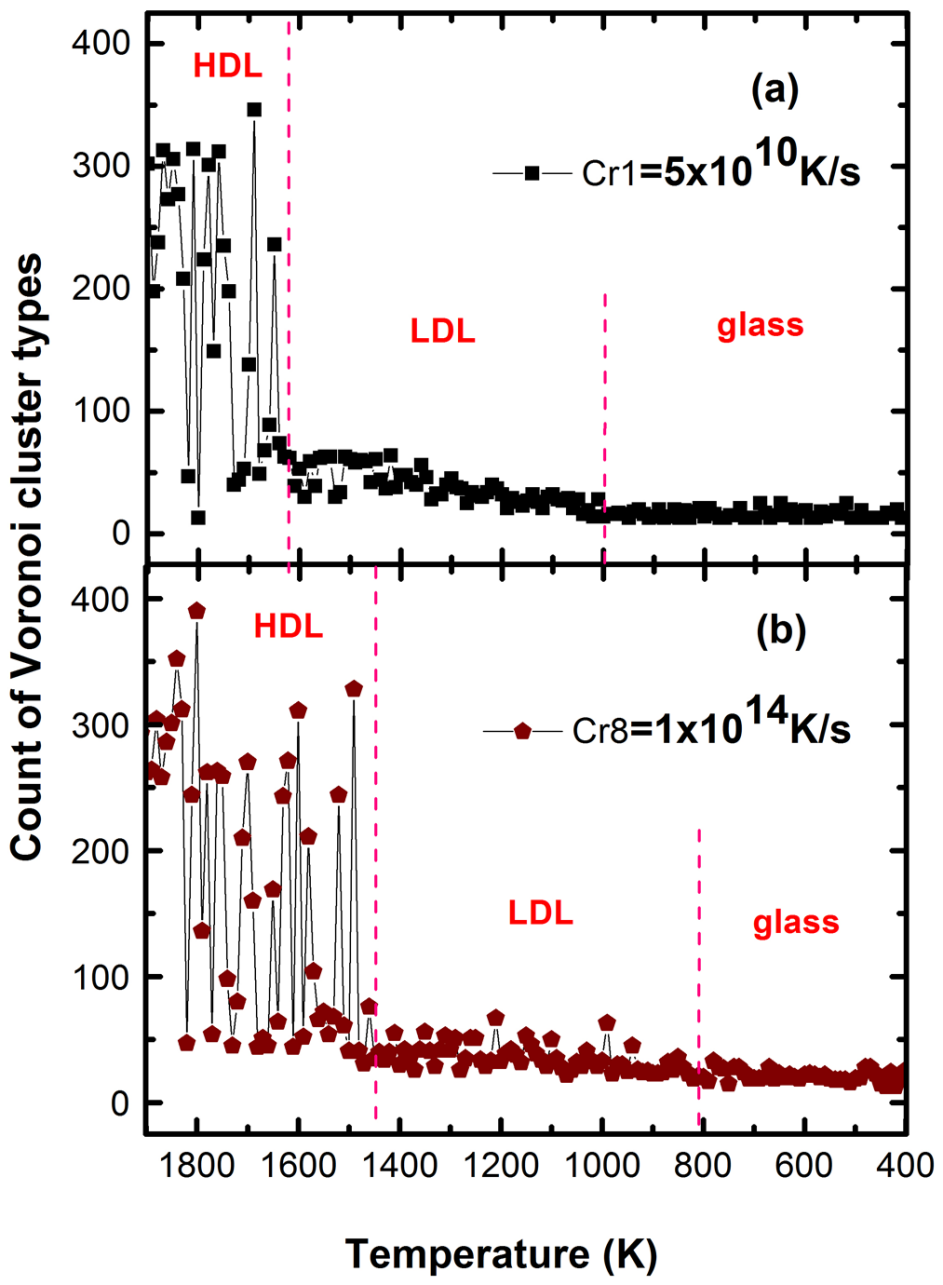
Temperature dependence of the number of the total voronoi cluster types upon two cooling rates. The types of the clusters are much abundant in high density liquids.

Voronoi polyhedral analyses is a geometrical spatial method and, can identify the unknown structures and give some statistics information about individual atoms such as the atomic coordination number and 3-D space shared by the central atom and all its neighbors. The Voronoi polyhedron method has been proven to be effective for the analyses of local atomic environment^31^ and the volume of cluster^32^ in liquids and glasses. The Voronoi index is expressed as <*n_1_,n_2_,n_3_,n_4_*>, where *n_i_* denotes the number of *i*-edged faces of the Voronoi polyhedrons^33^,^34^,^35^. Figure 5 shows the voronoi structure obtained from four typical cooling rates (a) cr1 = 5 × 10^10^ K/s, (b) Cr3 = 5 × 10^11^ K/s, (c) Cr6 = 1 × 10^13^ K/s and (d) Cr8 = 1 × 10^14^ K/s. Among the high temperature region (above ~1600 K), because of severe thermal motions, the types of the clusters are so diverse that the population is nearly averaged for each cluster. The maxnium fraction is less than 10% and none of them is dominant in HDL. The main structure is eight-fold coordination structure <0, 4, 4, 0> and the number of the types of the Voronoi clusters could reach up to 300–400 or more (Figure 6). As temperature is cooled down and pass by LLCT, that is, below 1600 K or 1500 K, the population of clusters in the supercooled silicon changed abruptly. The types of the Voronoi cluster decreases to about 40–50 (Figure 6), and the fraction of the low-coordination structure <0, 5, 2, 0>, <1, 3, 3, 0>, <2, 2, 2, 0> and <0, 6, 0, 0> reaches ~15%. Some outstanding structures, such as five-fold coordination structure <2, 3, 0, 0>, six-fold coordination structure <2, 2, 2, 0> and <0, 6, 0, 0>, rise sharply. The five-fold coordination structure <2, 3, 0, 0> becomes dominant in LDL. The distinct microstructure should correspond to the occurance of the LLPT in the supercooled liquid silicon. Therefore, the LLPT is associated with the particle aggregation behavior of five-fold or six-fold coordination structure from higher coordination structure.

### Relationship between LLPT and glassy silicon

As the LDL is cooled to glass state (below ~1000 K), significant difference in microstructure can be seen from the Figure 5(a)–(d). For the lowest cooling rate Cr1 (Figure 5a), the four-fold structure <4, 0, 0, 0> reaches 60% and becomes dominant instead of the five-fold structure <2, 3, 0, 0>. In constract, the fraction of five-fold structure <2, 3, 0, 0> slightly declines. The final solid is composed of about 70% <4, 0, 0, 0> and 25%<2, 3, 0, 0>. For the lower cooling rate Cr3 (Figure 5b), the four-fold structure <4, 0, 0, 0> reaches 40% and has the same fraction as the five-fold structure <2, 3, 0, 0>. For the faster cooling rate Cr6 (Figure 5c), the five-fold structure <2, 3, 0, 0> keep dominant and reaches 45% and the four-fold structure <4, 0, 0, 0> amounts to 25%. The other structures, such as six-fold structure <0, 6, 0, 0> and <2, 2, 2, 2>, keep the similar fraction as in LDL. For the fastest cooling rate Cr8 (Figure 5d), the fraction of the five-fold structure <2, 3, 0, 0> keeps increasing during cooling. Different from other cooling rates, the fraction of the four-fold structure <4, 0, 0, 0> increases sluggishly and the maximum fraction is only 20%. The six-fold structure <0, 6, 0, 0> and <2, 2, 2, 2> remain stable. The other six-fold structures which exist in LDL slightly decrease. It appears there is significant difference in glass microstructure for various cooling processes, and the main difference occurs in the percentage of the five-fold structure <2, 3, 0, 0> and the four-fold structure <4, 0, 0, 0>. Whereas the cooling rates have the marked influence on the <4, 0, 0, 0> clusters. the five-fold structure <2, 3, 0, 0> basically remains one of primary components in the glassy silicon.

In our previous wrok^33^, it was shown that the <4, 0, 0, 0> is the diamond crystal structure and existed in the crystal silicon. This structure makes the crystal silicon show semiconducting behaviors. The <2, 3, 0, 0> is similar to the diamond but it has an extra atom. Both of the two structures are sketched in the insets of Figure 5 (a) and Figure 5(b). Acoording to the PCFs at 600 K in Figure 3, the split in the second peak occurs from faster process Cr4 to the fastest process Cr8, as commonly observed in metallic glasses. The results are consistent with the distribution of coordination number in Figure 2, and the Voronoi structure in the glass in Figure 5(a)–(d).

## Discussion

The glassy silicon can be concluded to have three types of amorphous structures: <4,0,0,0>-based glass, <2,3,0,0>-based glass and <4,0,0,0> – <2,3,0,0>- mixture glass. The <2,3,0,0> is the main component in the LDL. The <4,0,0,0> can be transformed from the <2,3,0,0> as an atom is released^33^. The <4,0,0,0>-based glass is composed mainly of the four-fold structure <4, 0, 0, 0> and the less five-fold structure <2, 3, 0, 0>. So the solid shows the semiconducting behavior just like diamond crystal structure silicon. It can be achieved via the slower cooling process. The <2,3,0,0>-based glass is composed of the more five-fold structure <2, 3, 0, 0> and the less four-fold structure <4,0,0,0>. The solid consequently has the metallic behavior inherited from the liquid. It can be achieved via the faster cooling process. The <4,0,0,0> – <2,3,0,0>- mixture glass is composed of <4, 0, 0, 0> and <2, 3, 0, 0> equally, and the explanation of the properties is not clear. For the case of the fastest cooling process (Cr8 = 1 × 10^14^ K/s), because of the short relaxation time, the main structures in LDL such as six-fold structure <0, 6, 0, 0> and <2, 2, 2, 2> are preserved in the final glass. In contrast, the high coordination structures in HDL always transfer to the 5 or 6 coordination structure, including <2, 3, 0, 0>, <0, 6, 0, 0> and <2, 2, 2, 2>. The HDL structure can not be trapped in the final glass in the present studies.

## Methods

A series of rapid cooling process of liquid silicon is simulated by using LAMMPS code^36^. The initial structure of liquid silicon is obtained by enough relaxation from diamond silicon under 3000 K temperature. The system includes 4096 silicon atoms with periodic boundary condition (PBC). The atomic interactions are described using the Stillinger–Weber potential (SW potential)^37^, which is known to reproduce qualitatively the behavior of silicon well^33^,^38^. To obtain the supercooled liquid, the initial equilibrium liquid drop some temperature, such as 10 K, and repeat a relaxation under constant number, pressure, temperature (NPT) ensemble. The Velocity-Verlet algorithm is used with a integration time step of 1 fs (5 × 10^−15^ seconds). The pressure are controlled at 0 GPa via Nose-Hoover barostat. Run lengths range from 0.2 ns (500 000 steps) to 0.0001 ns (100 steps) to adjust the cooling rate from 5 × 10^10^ K/s to 1 × 10^14^ K/s. Obviously, the liquid system is hardly to reach the equilibrium and it is under supercooled state.

## Author Contributions

S.Z. and R.L. designed research and wrote manuscript. S.Z., L.Q. and S.Z. performed the simulations. L.W., X.Z. and M.M. analyzed the results and contributed to discussion. All authors reviewed the manuscript.
